# Morpheme Position Coding in Reading Development as Explored With a Letter Search Task

**DOI:** 10.5334/joc.153

**Published:** 2021-02-17

**Authors:** Jana Hasenäcker, Maria Ktori, Davide Crepaldi

**Affiliations:** 1 International School for Advanced Studies (SISSA), Trieste, Italy

**Keywords:** Visual word processing: Visual search, Development of cognition: Learning

## Abstract

Suffixes have been shown to be recognized as units of processing in visual word recognition and their identification has been argued to be position-specific in skilled adult readers: in lexical decision tasks suffixes are automatically identified at word endings, but not at word beginnings. The present study set out to investigate whether position-specific coding can be detected with a letter search task and whether children already code suffixes as position-specific units. A preregistered experiment was conducted in Italian in which 3rd-graders, 5th-graders, and adults had to detect a target letter that was either contained in the suffix of a pseudoword (e.g., *S* in *flag**ish***) or in a non-suffix control (e.g., *S* in *flag**osh***). To investigate sensitivity to position, letters also had to be detected in suffixes and non-suffixes placed in reversed position, that is in the beginning of pseudowords (e.g., *S* in ***ish**flag* vs. ***osh**flag*). Results suggested position-specific processing differences between suffixes and non-suffixes that develop throughout reading development. However, some effects were weak and only partially compatible with the hypotheses. Therefore, a second experiment was conducted. The effects of position-specific suffix identification could not be replicated. A combined analysis additionally using a Bayesian approach indicated no processing differences between suffixes and non-suffixes in our task. We discuss potential interpretations and the possibility of letter search being unsuited to investigate morpheme processing. We connect our example of failed self-replication to the current discussion about the replication crisis in psychology and the lesson psycholinguistics can learn.

The nature of the units that readers use when reading printed words has been one of the most important questions of reading research over the last few decades. There has been an ongoing debate on whether readers process letters individually or chunk them into higher-order units like graphemes, syllables, or morphemes. With regard to the latter, an accumulation of recent evidence suggests that skilled adult readers automatically decompose morphologically complex words into stem and affix (for a review see Amenta & Crepaldi, 2012). Some studies have indicated that this decomposition mechanism is sensitive to position: typical morphological effects, such as morpheme interference or morphological priming, disappear if the orthographic string, which represents a suffix (e.g., *–ful*), occurs in the beginning rather than at the end of a pseudoword (e.g., ***ful**gas*^1^ vs. ***gas**ful*), likely because it is not recognized as a morpheme in word-initial position (Crepaldi, Hemsworth, Davis, & Rastle, 2015; Crepaldi, Rastle, & Davis, 2010). This position-specific coding of morphemes in the visual identification system might support the efficiency of affix detection in complex words. However, it is unknown whether children are already sensitive to position in the identification of affixes as processing units.

Evidence for morphemes as units of processing in reading comes mainly from two phenomena: the *morpheme interference effect* and morphological priming effects. The morpheme interference effect describes the observation that affixed pseudowords (e.g., *gas**ful***) are harder to reject than non-affixed pseudowords (e.g., *gas**fil***) in a lexical decision task (e.g., Burani, Marcolini, & Stella, 2002; Crepaldi et al., 2010). Studies using masked morphological priming have shown that suffixed words (e.g., *read**er***) and also pseudosuffixed words (e.g., *corn**er***) facilitate the processing of the embedded stem (*read* and *corn*, respectively) regardless of whether it is morphologically related, while this is not true for words with non-suffix endings (e.g., *spin**ach**-spin*, for a review see Rastle & Davis, 2008). This has been interpreted as evidence that affixes are automatically detected on the basis of orthography and “stripped off” such that the stem is activated (e.g., Taft & Forster, 1975). Moreover, suffixed nonword primes facilitate the recognition of target words with the same suffix (e.g., *sheet**er**-teach**er***), while this is not the case for non-suffix endings (e.g., *sport**el**-broth**el***) (Crepaldi et al., 2015).

Recently, Beyersmann, Ziegler, & Grainger (2015) used a different task to test the hypothesis that suffixes (and prefixes) are processed as reading units. They employed a letter search task with affixed and non-affixed pseudowords and found that participants took longer to detect a target letter when it was part of a suffix in a pseudoword (e.g., *R* in *film**ure***) than when it was part of a non-suffix ending (e.g., *R* in *film**ire***). This supports the automatic identification of suffixes in visual word recognition. However, no difference was found between prefixed and nonprefixed pseudowords (e.g., *R* in ***pro**point* vs. *R* in ***cro**point*). This approach stands in a long tradition of using letter search tasks to examine perceptual units of different sizes in the reading system. Interestingly, these studies support different theoretical accounts, depending on the unit under examination. Letter search studies on the level of words suggest that letters are consistently easier to detect when they appear within existing units: a letter is easier to detect in a word (e.g., *K* in *work*) as compared to a pseudoword (e.g., *K* in *wosk*) or an unpronounceable string of letters (e.g., Reicher, 1969; Wheeler, 1970). This was typically explained by lexical feedback in the classic interactive activation framework — word nodes get activated and send feedback to the letter level, which results in a faster processing of these units (e.g., Coltheart et al., 2001; McClelland & Rumelhart, 1981; Paap, Newsome, McDonald, & Schvaneveldt, 1982). Most morphological models assume very similar dynamics and architectures (e.g., Crepaldi et al., 2010). Thus, if suffixes are represented as units, a similar facilitation should be seen for letter detection in suffixes as compared to non-suffixes. The contradicting hypothesis, namely that letters are *harder* to detect in a suffix as compared to a non-suffix, was supported by Beyersmann et al. (2015). The authors reason that the *chunking* of suffixes inhibits the activation of the single letters within that unit, thus hampering the detection of single letters in suffixes but not in non-suffixes (see also Davis, 1999; Grainger & Ziegler, 2011). This follows seminal evidence on letter detection difficulties in suffixes by Drewnowski and Healy (1980). In their study, participants read connected texts and were instructed to circle the letter *n* whenever it appears. Participants missed significantly more occurrences of *n* in *–ing*, and especially so if *–ing* was a suffix (e.g., as in *hav**ing*** vs. *dur**ing***). This was interpreted in terms of *unitization*, such that supraletter units can be activated without the complete identification of their component letters (for a review see also Healy, 1994). Later letter search studies with single word presentation found similar effects also on the level of syllables and graphemes. Rey, Ziegler, and Jacobs (2000) report that a letter is harder to detect in a multi-letter grapheme (e.g., *A* in *beach*) as compared to a single-letter grapheme (e.g., *A* in *place*) (see also Commissaire & Casalis, 2018). Similarly, Brand, Giroux, Puijalon, and Rey (2007) report that letters are harder to detect in multi-letter syllable onsets (e.g., *L* in *tablier*) than single-letter syllable onsets (e.g. *L* in *horloge*).

Importantly, some studies on morphological processing indicate that suffix identification is position-specific: letter strings resembling suffixes (e.g., *-ful*) are identified automatically as morphemes only at word endings, but not at word beginnings, where they do not typically occur. The morpheme interference effect does not emerge for pseudowords with a reversed order of stem and suffix (e.g., ***ful**gas*) (Crepaldi et al., 2010). Also, when the order of stem and suffix in primes is reversed, the recognition of words with the same suffix is not facilitated (e.g., ***er**sheet-teach**er***) (Crepaldi et al., 2015). Indeed, Drewsnoski and Healy (1980) already put forward the idea that the letter search effect for suffixes is position-specific. However, most of the major models of morphological processing do not specify a functional role of morpheme position coding (e.g., Rastle & New, 2008). An exception to this is the fine-grained route of processing that Grainger and Ziegler (2011) and Grainger and Beyersmann (2017) assume, which operates on ordered letter strings. However, their proposal pertains more to the order of the letters within a morpheme than the order of the morphemes within the complex words.

Effects of morphological decomposition have been shown also for children, implying that suffixes are important units already in reading development. The morpheme interference effect in lexical decision has been shown to be present already in 3^rd^ grade Italian as well as French readers (Burani et al., 2002; Casalis, Quémart, & Duncan, 2015; Quémart, Casalis, & Duncan, 2012). Also, facilitation from suffixes in the reading of real words has been shown as early as in 2^nd^ grade in several languages (English: Beyersmann, Castles, & Coltheart, 2011; French: Casalis et al., 2015; Quémart et al., 2012; German: Hasenäcker, Schröter, & Schroeder, 2017; Italian: Burani et al., 2002; Burani, Marcolini, De Luca, & Zoccolotti, 2008; Marcolini, Traficante, Zoccolotti, & Burani, 2011). These studies leave open at what level of processing suffixes play a role – at lower visuo-orthographic or at higher lexico-semantic levels. Results from masked suffix priming in children overall suggest automatic activation (English: Beyersmann, Castles, & Coltheart, 2012; French: Beyersmann, Grainger, Casalis, & Ziegler, 2015; Casalis, Dusautoir, Colé, & Ducrot, 2009; Quémart, Casalis, & Colé, 2011; German: Hasenäcker, Beyersmann, & Schroeder, 2016, 2020), but are mixed with regard to the contribution of orthographic, morphological or semantic processes. How exactly the underlying mechanism recognizes stems and affixes is still hotly debated. Therefore, it appears useful to test morphological processing with other tasks that tap into early visuo-orthographic identification of suffixes.

To our knowledge, only one recent study has explored developing readers’ sensitivity to morphemes at early visuo-orthographic stages using a letter search task. Antzaka, Acha, Carreiras, and Lallier (2020) compared letter search in stems and suffixes in Basque 4^th^-grade children. In contrast to the letter search studies with adults, they did not find a difference in letter search performance based on the presence of morphemes in the string. However, their items also differed from the ones used by Beyersmann et al. (2015): Antzaka et al. (2020) used morphologically complex pseudowords that included either a stem or a suffix (+stem–suffix, –stem+suffix) as well as morphologically simplex (–stem–suffix) pseudowords. By contrast, all of the items of Beyersmann et al. (2015) encompassed a real stem and either an affix or a non-affix (+stem+suffix, +stem–suffix). Moreover, Antzaka et al. (2020) did not match the frequency of the suffix and non–suffix endings. It is thus unclear whether children do not yet show the same effects as adults or the lack of the effects is due to the stimuli used.

With regard to suffix position, it is completely unclear whether suffix identification is already position-specific in children, as no study has investigated this issue before. On the one hand, children already exploit morphological structure in reading, but show sensitivity to prefixes, suffixes and stems at different time points during their development: while they use stems as processing units as early as in grade 2, they start using suffixes only one year later in grade 3, and prefixes another year later in grade 4 (Hasenäcker et al., 2017). This developmental difference between prefixes, suffixes and stems might be related to positional constraints, depending on where in the word the units occur. Even beginning readers might already code suffixes in a position-specific manner, because it additionally aids their detection. Word recognition is easier when the child can draw on top-down knowledge about where to expect a suffix. On the other hand, position-specific coding can be expected to arise from distributional properties within the language, that is, children learn from exposure where a certain morpheme most likely appears. Beginning readers’ experience with written words, however, might still be too limited to pick up such orthographic regularities and automatically use them in word recognition. Dawson, Rastle, and Ricketts (2018) argue that some important changes in morphological processing, in particular concerning the automaticity of suffix chunking and suffix recognition in pseudowords, occur as late as in adolescence. It is thus an open question whether suffixes are position-specific perceptual units used by elementary school children.

In the present study, we use a letter-search task with suffixed and nonsuffixed pseudowords, as previously employed by Beyersmann et al. (2015), with Italian readers. In extension to Beyersmann et al. (2015), we follow two goals: 1) to test position-specificity of suffix identification in skilled readers, 2) to test whether suffixes are perceptual (and position–specific) units already in reading development. Participants in our study were asked to detect a target letter, which is either contained in the suffix of a pseudoword (e.g., *S* in *flag**ish***) or in a non-suffix control (e.g., *S* in *flag**osh***). Based on the evidence in the literature showing that letter detection is influenced by the status of the letter string, we can assume that the letter search task should be sensitive to the morphological status of the stimuli, at least in the case of skilled readers: letters (e.g., *S*) belonging to units identified as suffixes (e.g., *ish* in *flag**ish***) will behave differently from letters lacking this feature (e.g., *S* in *flag**osh***). Based on theoretical considerations and previous empirical evidence, there are two opposing hypotheses for the direction of the effect, that is, whether letter detection is easier or more difficult in suffixes as compared to the non-suffix control condition. Critically, both hypotheses predict a difference between suffixes and nonsuffixes. In order to investigate sensitivity to morpheme position, we extend the paradigm to pseudowords with reversed morphemes: letters also have to be detected in affixes in their untypical position (e.g., *S* in ***ish**flag*) with nonsuffixed pseudowords as controls (e.g., *S* in ***osh**flag*). Suffixes in the reversed condition should not be automatically perceived as units. Consequently, any effect of affix status should disappear in the reversed condition, if affixes are identified in a position-specific fashion. Furthermore, we were interested to see whether developing readers already show the same pattern of processing as adults or whether their exposure to suffixes as position-specific units has been too limited to show effects in visual perception in reading, as the study by Antzaka et al. (2020) suggests.

In the following, we will present the study as two experiments, analyzed separately first, followed by an aggregated analysis. The first experiment of the study was preregistered on the Open Science Framework prior to data collection. The preregistration is available on the project side: *https://osf.io/yvtna/*. In this preregistration, we specified the hypotheses outlined above, as well as the exact method of data collection and analysis, including the sample size. After data collection and analysis following our preregistration, the results were inconclusive and were not clearly reconcilable with either of our hypotheses. Therefore, in the second experiment, we repeated the study with approximately the same number of new participants in an attempt to self-replicate. For completeness and to make best use of the bigger sample size that the two experiments span together, we also present an analysis on the data collapsed across studies.

## Experiment 1

### Method

#### Participants

Forty-five native Italian-speaking children attending 3^rd^ grade and 41 children attending 5^th^ grade of an Italian primary school were tested either in their school, in their after-school care center, or in our laboratories. In addition, 39 native Italian-speaking adults participated in the experiment in our laboratory for monetary compensation. Written consent was obtained prior to the experiment from the adults and from the parents in the case of the children. Four children (three 3^rd^-graders, one 5^th^-grader) were excluded due to language-related problems, as indicated by their parents. None of the other participants had a diagnosis of any reading-related or cognitive disability and all of them had normal or corrected-to-normal vision. Furthermore, twelve participants were excluded (four 3^rd^-graders, seven 5^th^-graders, one adult), because their performance was below chance level (>60% errors), indicating that they did not do the task correctly as they were instructed to.^2^ This left us with data from 38 3^rd^-graders (M_Age_ = 8.11, SD_Age_ = 0.38, 24 girls), 33 5^th^-graders (M_Age_ = 10.12, SD_Age_ = 0.33, 19 girls), and 38 adults (M_Age_ = 23.74, SD_Age_ = 3.45, 24 female).

#### Materials

To build the target stimuli, we chose eight different highly frequent Italian suffixes, of which half were 3-letters long (-*ale*, -*uto*, -*ame*, -*oso*), and half were 4-letters long (-*enza*, -*ismo*, -*ario*, -*ista*) and eight different non-suffix endings that were of comparable frequency and length as the suffixes and differed from the suffixes by only one letter (-*ole*, -*oto*, -*eme*, -*eso*, and -*enta*, -*isco*, -*arlo*, -*osta*). We matched suffixes and non-suffix endings on frequency in order to avoid that any potential effects could arise due to differences in familiarity with the orthographic string, instead of their morphological status. Suffix and non-suffix frequencies were calculated by summing the occurrences of the respective tri- or four-grams in word-final position using the subtlex-it database (Crepaldi, Keuleers, Mandera, & Brysbaert, 2013). Mean frequencies (log10 of total occurrences) for suffixes were 4.92 (*SD* = 0.52, range = 4.12–5.60) and for non-suffixes 4.80 (*SD* = 0.24, range = 4.58–5.20). We created pronounceable pseudowords by combining each of these suffix and non-suffix pairs with eight different stems that were 4–5 letters long and had a mean frequency (log10) of 3.93 (*SD* = 0.44, range = 3.23–4.96). Note that the vowel at the end of the stem needs to be dropped in Italian to adhere to morpho-phonological rules when the stem combines with a suffix or non-suffix ending (e.g., *libro+oso = libr**oso*** and *libro+eso=libr**eso***). To create the reversed condition, we changed the order of the suffixes/non-suffixes and stems (e.g., ***oso**libro* and ***eso**libro*). In this case, the vowel is not dropped from the stem in accordance with Italian morpho-phonology. Due to the vowel-drop feature and the syllabic structure of Italian, the stimuli in the reversed condition were always one letter longer than in the regular condition in order to keep the well-formedness of the pseudowords. The second letter of a suffix/non-suffix was always used as the target letter (e.g., *S* for *libr**oso**, libr**eso, oso**libro, **eso**libro*). Four counter-balanced lists were created using a Latin Square design, such that each participant is presented with each stem only in one combination, being exposed to 64 target stimuli (yes-response trials) in total, seeing equally frequently each of the four conditions (i.e., 16 trials per condition).

In addition, to create target-absent trials (no-responses), 64 pseudowords were constructed by following the same logic but using eight different suffixes (-*one*, -*ese*, -*ota*, -*ino*, and -*anza*, -*iere*, -*azzo*, -*ella*) and eight different non-suffix endings (-*ene*, -*ase*, -*ita*, -*ono*, and -*anda*, -*iete*, -*ezzo*, -*alla*) matched on frequency to the ones chosen for the target stimuli. Those were combined with different stems matched on frequency, length in letters and OLD20 to the stems of the target stimuli. The target letters were chosen such that they were not present in the pseudowords (e.g., *C* for *vitone*).

In order to balance out target letter position and to include pseudowords without a morphological structure, 64 additional filler trials were created by choosing monomorphemic Italian words (e.g., *fortuna*) and changing one letter to create pseudowords (e.g., *fartuna*). Half of the filler trials contained the target letter (target-present fillers) eliciting yes-responses and half did not contain the target letter (target-absent fillers) eliciting no-responses. The position of the target letters in target-present fillers was varied throughout the pseudoword. The complete set of target stimuli is presented in the Supplementary Material (Table S1).

#### Procedure

The experiment was run using the PsychoPy software (Peirce, 2007). All stimuli were presented in white font on black background. Each trial was initiated by a fixation cross presented centrally for 1000ms. This was followed by the target letter in uppercase for 500ms, which was immediately replaced by the pseudoword presented in lowercase for 500ms. Thereafter, the screen remained blank until participants’ response or a response time-out of 2000ms after pseudoword onset. Participants’ response or elapse of the time-out was followed by a 2000ms interval with a blank screen until the next trial was automatically initiated. Participants were instructed to decide as quickly and as correctly as possible whether the target letter was present in the pseudoword or not. They were asked to indicate the presence by pressing the K button on the keyboard (marked green) and indicate the absence by pressing the D button (marked red) with their index fingers. Response times and accuracies were registered. The 192 trials were randomly divided into three experimental blocks of 64 trials each. Between experimental blocks, participants were given a break. Prior to the first experimental block, participants had ten practice trials to get accustomed to the task.

#### Results

**Figure 1:**
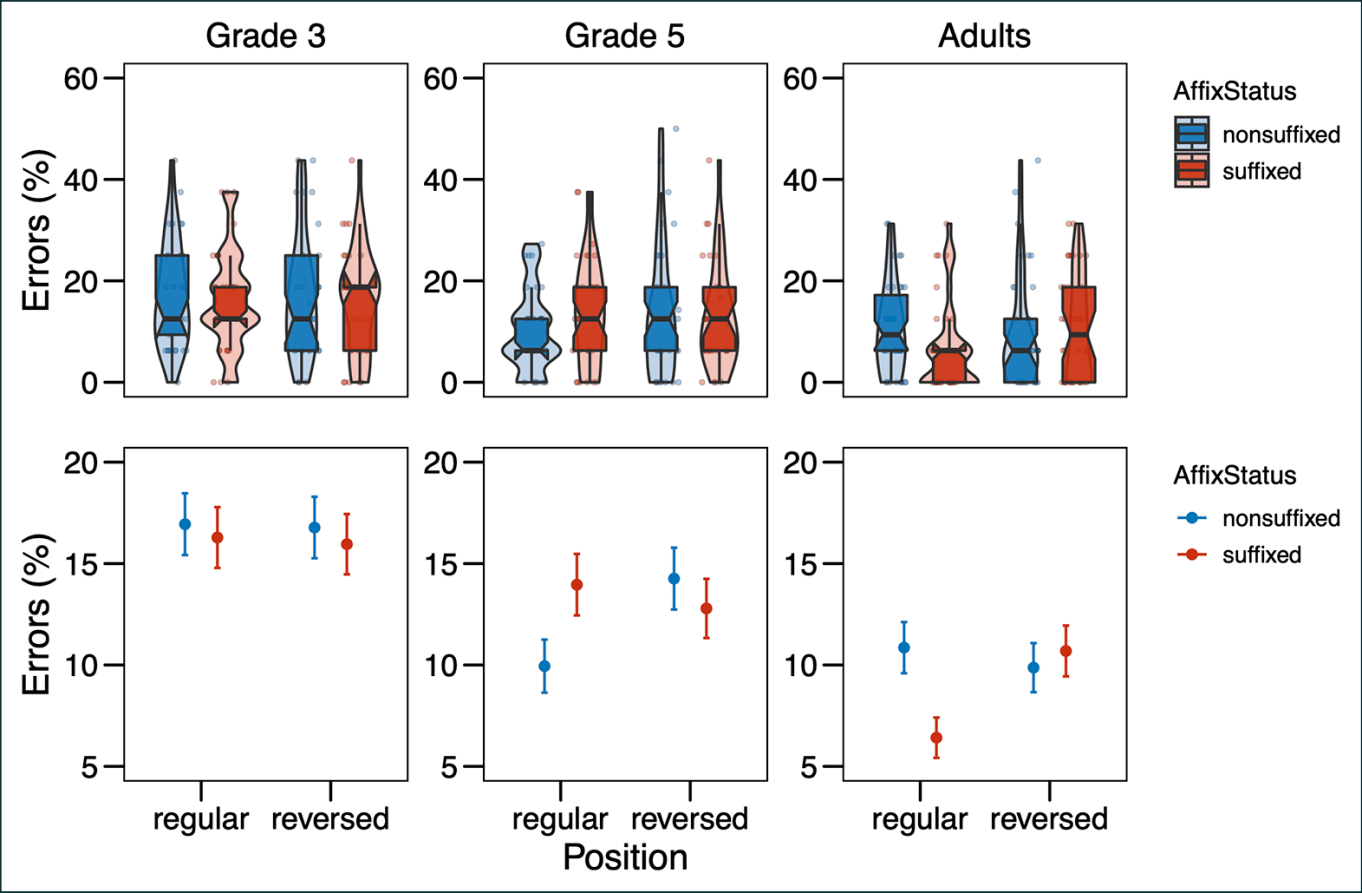
Error rates (in %) from Experiment 1 in the different conditions (Affix Status × Position) by age group. The upper row shows the distribution of the data, with boxplots indicating medians and interquartile ranges; the points represent by-subject means. In the bottom row, the points refer to the means by condition, while the error bars show the standard errors of the mean calculated at the trial-level.

**Figure 2:**
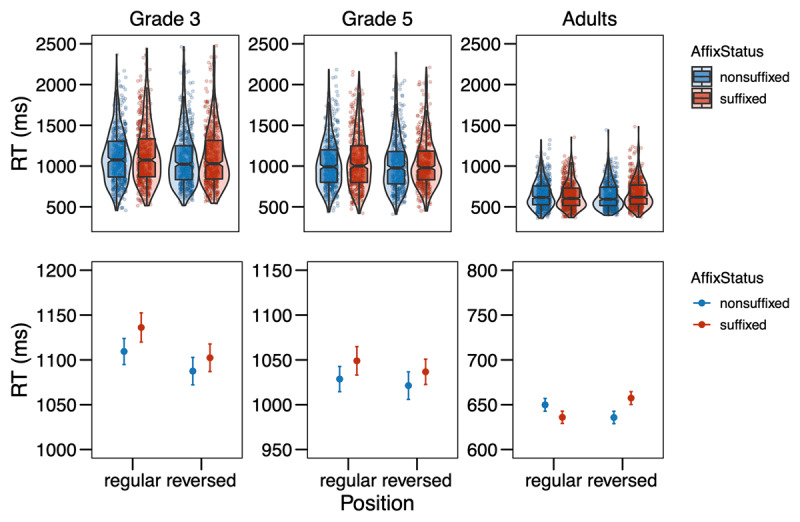
Response times (in ms) from Experiment 1 in the different conditions (Affix Status × Position) by age group. The upper row shows the distribution of the data, with boxplots indicating medians and interquartile ranges; the points represent single data points. In the bottom row, the points refer to the means by condition, while the error bars show the standard errors of the mean calculated at the trial-level. Note that the scales are equal across grades in the upper row, but range in the bottom row.

All data analyses were carried out on target-present trials using the statistical software R, following the preregistered procedure. Data and scripts are available at *https://osf.io/yvtna/*. Means of error rates and response times are presented in ***Table 1*** and ***Figures 1*** and ***2***, respectively. Error rates and response time data were analyzed separately using (generalized) linear mixed-effects modeling with the lme4 package (Bates, Maechler, Bolker, & Walker, 2015). As stated in the preregistration, a forward model selection procedure was used starting with our variables of interest, namely Affix Status (suffix vs. non-suffix), Position (regular vs. reversed), and Grade (3 vs. 5 vs. Adults) and their interactions as fixed effects as well as Participant and Item as random effects. Trial Order and Target Letter Identity and their interaction with Grade were added as fixed effects when model comparison suggested that they significantly improved the model fit.^3^ In the following, we present results for the overall effects tests using contrast coding and Type III sum of squares of the final models selected with this procedure. Post-hoc comparisons were made using the emmeans package (Lenth, 2019) to decompose significant interactions.

**Table 1:** Means and ΔMeans of Error Rates and Response Times in Experiment 1 (Standard Deviations in Parentheses).


POSITION	REGULAR			REVERSED		

AFFIX STATUS	SUFFIXED	NONSUFFIXED	Δ_(NON-SUFF)_	SUFFIXED	NONSUFFIXED	Δ_(NON-SUFF)_

Error Rates (in %)						

Grade 3	16.28 (1.50)	16.94 (1.52)	0.66	15.95 (1.49)	16.78 (1.52)	0.83

Grade 5	13.96 (1.52)	9.94 (1.31)	–4.02*	12.79 (1.46)	14.26 (1.53)	1.47

Adults	6.41 (0.99)	10.86 (1.26)	4.45*	10.69 (1.25)	9.87 (1.21)	–0.82

Response Times (in ms)						

Grade 3	1136 (363)	1109 (323)	–27	1102 (345)	1088 (340)	–14

Grade 5	1049 (332)	1029 (302)	–20	1037 (300)	1021 (324)	–16

Adults	636 (161)	650 (165)	14	657 (166)	636 (160)	–21*


* Significant difference (*p* < .05) according to the reported linear mixed-effects model.

For the analysis of the error rates, the final model included Affix Status, Position, Grade, and their interactions as fixed effects, as well as Trial Order and an interaction of Target Letter Identity and Grade as covariates, and Participant and Item as random effects. The model revealed significant effects of the covariates Trial Order (χ*^2^* = 14.04, *p* < .001) and Target Letter Identity × Grade (χ*^2^* = 123.38, *p* < .001). For the variables of interest, there was a main effect of Grade (χ*^2^* = 21.92, *p* < .001) and a marginally significant main effect of Position (χ*^2^* = 2.72, *p* = .099). Importantly, these main effects were involved in a three-way interaction of Affix Status, Position, and Grade (χ^2^ = 9.42, *p* = .009). Decomposing this three-way interaction reveals that for 3^rd^-graders, there was no effect of Affix Status, neither in the regular (*z* = –0.373, *p* = 0.709), nor in the reversed position (*z* = –0.289, *p* = 0.773). For 5^th^-graders, there was a weak effect for Affix Status in the regular position with a tendency for letters in non-suffixes to be recognized more correctly as compared to suffixes (*z* = 2.035, *p* = 0.042), and no effect in the reversed position (*z* = –0.637, *p* = 0.524). 3^rd^- and 5^th^-graders differed marginally in their effects of Affix Status in the regular position (*z* = –1.834, *p* = 0.066), but not in the reversed position (*z* = 0.296, *p* = 0.767). For adults, there was a significant effect of Affix Status in the regular position with letters being recognized more correctly in suffixes as compared to non-suffixes (*z* = –2.732, *p* = 0.006), but no such effect in the reversed position (*z* = 0.511, *p* = 0.610). 5^th^-graders and adults differed significantly in their effects of Affix Status in the regular position (*z* = 3.412, *p* < 0.001), but not in the reversed position (*z* = –0.816, *p* = 0.414), and the same was found for 3^rd^-graders and adults (regular: *z* = 1.987, *p* = 0.047; reversed: *z* = –0.583, *p* = 0.560). The full model output (following the guidelines by Meteyard & Davies, 2020) is presented in the Supplementary Material Table S2.

For the analysis of the response times, incorrect responses were removed (3^rd^ grade: 16.49%, 5^th^ grade: 12.74%, adults: 9.46%), then response times below 200ms were removed as false alarms (3^rd^ grade: 0.05%, 5^th^ grade: 0.00%, adults: 0.00%). Next, response times were logarithmically transformed and further outliers were trimmed by removing all data points with residuals exceeding 2.5 SD based on a simple model including only random effects (3^rd^ grade: 1.87%, 5^th^ grade: 1.59%, adults: 1.95%). Then models were fitted and selected as described above. The final model included Affix Status, Position, Grade, and their interactions as fixed effects, as well as interactions of Trial Order and Grade, and Target Letter Identity and Grade as covariates, and Participant and Item as random effects. The model revealed significant effects of the covariates Trial Order × Grade (χ*^2^* = 138.11, *p* < .001) and Target Letter Identity × Grade (χ*^2^* = 338.56, *p* < .001). More interestingly, for the variables of interest, we found a main effect of Grade (χ*^2^* = 194.10, *p* < .001), a main effect of Position (χ*^2^* = 4.73, *p* = .030), and a main effect of Affix Status (χ*^2^* = 3.95, *p* = .047). Those main effects entered into a two-way interaction between Grade and Position (χ*^2^* = 7.91, *p* = .019), indicating that responses were faster in the reversed position for 3^rd^-graders (*z* = 3.046, *p* = 0.002), but not for 5^th^-graders (*z* = 1.504, *p* = 0.133) and not for adults (*z* = –0.802, *p* = 0.423). Finally, there was a marginally significant three-way interaction between Affix Status, Position, and Grade (χ*^2^* = 5.88, *p* = .053). In order to investigate whether the three-way interaction is reliable, we applied model criticism to the initial model without covariates, excluding residuals above 2.5SD (Baayen & Milin, 2010). We then repeated the model selection process as described above. The final model was the same as before and the overall pattern of effects also remained the same with the exception that the main effect of Affix Status was only marginally significant now (χ*^2^* = 3.63, *p* = .057), whereas the three-way interaction was clearly significant (χ*^2^* = 8.55, *p* = .014). Decomposing the three-way interaction revealed that for 3^rd^-graders, there was no effect of Affix Status, neither in the regular (*z* = –1.582, *p* = 0.114), nor in the reversed position (*z* = 0.316, *p* = 0.752). For 5^th^-graders, there was also no effect for Affix Status in the regular position (*z* = –0.692, *p* = 0.489), but a marginal effect in the reversed position with a tendency for letters in non-suffixes to be recognized faster as compared to suffixes (*z* = –1.848, *p* = 0.065). 3^rd^- and 5^th^-graders did not differ in their effects of Affix Status neither in the regular position (*z* = –0.603, *p* = 0.547) nor in the reversed position (*z* = 1.599, *p* = 0.110). For adults, there was no effect of Affix Status in the regular position (*z* = 1.418, *p* = 0.156), but a significant effect in the reversed position with letter search being faster in non-suffixes as compared to suffixes (*z* = –2.411, *p* = 0.016). Adults did not differ from 5^th^-graders in their effects of Affix Status neither in the regular (*z* = –1.502, *p* = 0.133) nor in the reversed position (*z* = 0.255, *p* = 0.799). However, the effect of Affix Status did differ significantly between 3^rd^-graders and adults in the regular position (*z* = –2.185, *p* = 0.029) and marginally in the reversed position (*z* = 1.948, *p* = 0.051). The full model output is presented in the Supplementary Material Table S3.

#### Discussion

The results of Experiment 1 were not clear-cut in either of the directions we hypothesized in the pre-registration. The analysis of error rates suggests that for skilled adult readers, detection of letters is easier in suffixes as compared to non-suffixes in their regular position (*S* in *flagish* vs. *flagosh*). This is in line with literature on the word superiority effect, which has shown that letter search is faster in words (e.g., *K* in *work*) than pseudowords (e.g., *K* in *wosk*) (e.g., Reicher, 1969; Wheeler, 1970), suggesting top-down activation from words to the letter level as assumed by the classic interactive activation framework (e.g., Coltheart et al., 2001; McClelland & Rumelhart, 1981; Paap et al., 1982). This finding attests the idea that suffixes are processed as units similar to words. The pattern is not in line with the opposing hypothesis and data by Beyersmann et al. (2015), arguing that letter detection is harder in suffixes than non-suffixes, because the chunking of suffixes inhibits the activation of the single letters within that unit (see also Davis, 1999; Grainger & Ziegler, 2011).

Importantly with regard to the question whether suffixes are *position-specific* units, no difference in error rates was found between suffixes and non-suffixes in the reversed position (*S* in ***ish**flag* vs. ***osh**flag*). Thus, suffixes are automatically processed as units only when they are in their regular word-final position, but not at word beginnings, where they do not typically occur. This suggests position-specific coding of suffixes in line with Crepaldi et al. (2010, 2015). With regard to the different age groups, there was a clear developmental trend as the position-specific effect of affix status was only observed for adults, but not for children, indicating that children do not yet code suffixes as units. At first glance, this seems to be in contrast to masked priming studies that showed suffix priming effects in children (e.g., Beyersmann et al., 2012; Beyersmann et al., 2015; Casalis et al., 2009; Quémart et al., 2011; Hasenäcker et al., 2016), but recent theories of morphological processing have advocated for those effects arising based on the embedded stem rather than the affix (Grainger & Beyersmann, 2017), especially in reading development (cf. Hasenäcker et al., 2017), leaving open the possibility that representations of suffixes as units are not yet in place in elementary school, as the present study suggests.

In contrast to the error rate analysis, the analysis of the response times suggests that for skilled adult readers, there was no difference between letter detection in suffixes and non-suffixes in their regular position (*S* in *flag**ish*** vs. *flag**osh***), while there was a difference in the reversed position with letter detection taking longer in suffixes as compared to nonsuffixes (*S* in ***ish**flag* vs. ***osh**flag*). The same was seen as a trend in 5^th^-graders. This pattern of results is rather surprising and was predicted by neither of the two rivaling hypotheses that we presented in the beginning. However, without a significant effect in the regular position, the effect in the reversed position is difficult to interpret. A possible explanation could be that a suffix in the reversed, thus “wrong” position attracts much attention from skilled readers who associate suffixes with the word-final position. The longer reaction times are then an index of surprisal. This fits with the observation that children show a similar trend for longer response times to letters in suffixes in the regular condition as the adults show in the reversed condition (cf. ***Table 1***; ***Figure 2***): what is an index of surprisal in the adults, could reflect setting up representations of suffix representations in children. However, the explanation of such a surprisal effect is at odds with previous findings reviewed in the Introduction that suggest suffixes are only identified as such in their correct position (Crepaldi et al., 2010, 2015). Also, the three-way interaction between Affix Status, Position and Grade was rather weak and only reached significance after removing outliers via model criticism. The two-way interaction between Position and Grade seemed much more stable: 3^rd^-graders, but not 5^th^-graders or adults showed faster responses to the reversed than the regular condition, indicating that beginning readers search for the letter in a more serial fashion, scanning the nonword from left to right. A similar observation has been made by Antzaka et al. (2020), who found a clear left-to-right decrease in target letter detection performance in 4^th^-grade Basque-readers, but no boost in target letter detection due to the presence of suffixes. While the left-to-right bias is interesting in itself, unfortunately it does not allow any insights into processing mechanisms related to affixes.

In order to investigate the possibility that the inconclusive findings of Experiment 1 were due to noise and an insufficiently big sample size, we conducted the same experiment again with a similar number of new participants.

## Experiment 2

### Method

#### Participants

Forty-six native Italian-speaking children attending 3^rd^ grade and 45 children attending 5^th^ grade of an Italian primary school were tested in our laboratories as part of the citizen science program Brains@Work (Zampieri, 2018), in which school classes visit the institute to learn about science and take part in experiments. Moreover, 40 native Italian-speaking adults participated in the experiment in our laboratory for monetary compensation. Written consent was obtained prior to the experiment from the adults and from the parents in the case of the children. None of the participants had a diagnosis of any reading-related or cognitive disability and all of them had normal or corrected-to-normal vision, as indicated by the parents. Furthermore, 34 participants were excluded (20 3^rd^-graders, 14 5^th^-graders), because their performance was below chance level (>60% errors), indicating that they did not do the task as they were instructed to. This left us with data from 26 3^rd^-graders (M_Age_ = 8.35, SD_Age_ = 0.55, 10 girls), 31 5^th^-graders (M_Age_ = 10.32, SD_Age_ = 0.64, 12 girls), and 40 adults (M_Age_ = 23.83, SD_Age_ = 2.77, 29 female).

#### Materials and Procedure

The materials and procedure used were the same as in Experiment 1.

#### Results

**Figure 3:**
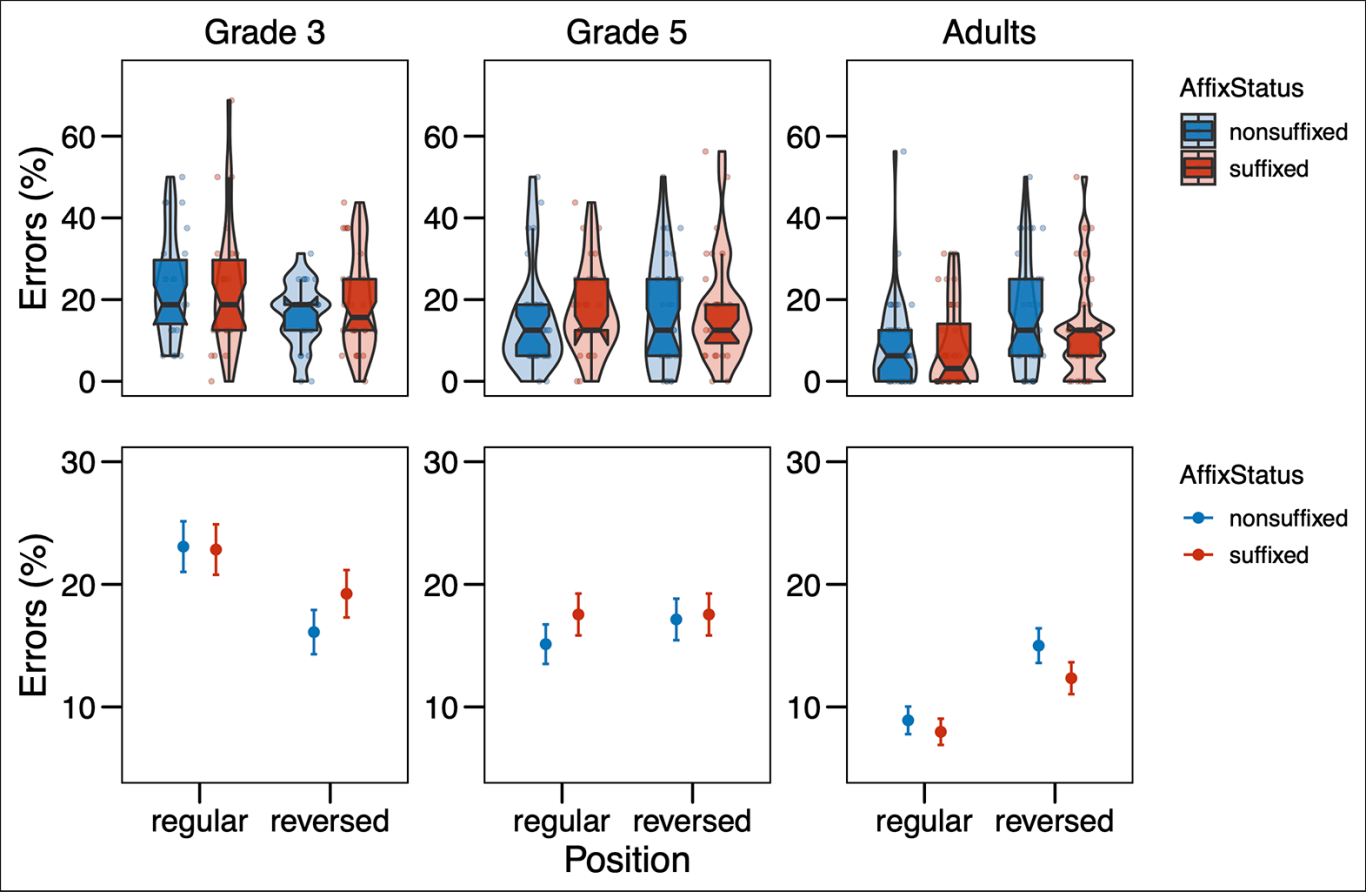
Error rates (in %) from Experiment 2 in the different conditions (Affix Status × Position) by age group. The upper row shows the distribution of the data, with boxplots indicating medians and interquartile ranges; the points represent by-subject means. In the bottom row, the points refer to the means by condition, while the error bars show the standard errors of the mean calculated at the trial-level.

**Figure 4:**
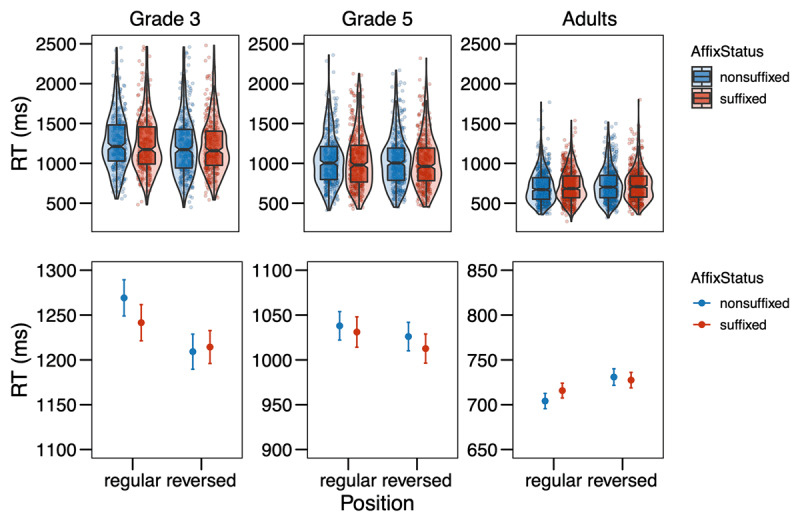
Response times (in ms) from Experiment 2 in the different conditions (Affix Status × Position) by age group. The upper row shows the distribution of the data, with boxplots indicating medians and interquartile ranges; the points represent single data points. In the bottom row, the points refer to the means by condition, while the error bars show the standard errors of the mean calculated at the trial-level. Note that the scales are equal across grades in the upper row, but range in the bottom row.

Means of error rates and response times from Experiment 2 are presented in ***Table 2*** and ***Figures 3*** and ***4***, respectively. Data were treated and analyzed as in Experiment 1 (data and scripts available at *https://osf.io/yvtna/*). However, instead of using a forward model selection procedure as in Experiment 1, we directly chose the models that turned out as final models in Experiment 1 in order to keep analyses consistent across experiments.

**Table 2:** Means and ΔMeans of Error Rates and Response Times in Experiment 2 (Standard Deviations in Parentheses).


POSITION	REGULAR			REVERSED		

AFFIX STATUS	SUFFIXED	NONSUFFIXED	Δ_(NON-SUFF)_	SUFFIXED	NONSUFFIXED	Δ_(NON-SUFF)_

Error Rates (in %)						

Grade 3	22.84 (2.06)	23.08 (2.07)	0.24	19.23 (1.93)	16.11 (1.80)	–3.12

Grade 5	17.54 (1.71)	15.12 (1.61)	–2.42	17.54 (1.71)	17.14 (1.69)	–0.40

Adults	7.97 (1.07)	8.91 (1.13)	0.94	12.34 (1.30)	15.00 (1.41)	2.66

Response Times (in ms)						

Grade 3	1242 (358)	1269 (352)	27	1214 (335)	1209 (362)	–5

Grade 5	1031 (340)	1038 (321)	7	1013 (322)	1026 (320)	13

Adults	716 (198)	704 (203)	–12	727 (202)	731 (213)	4


For the analysis of the error rates, the model revealed significant effects of the covariates Trial Order (χ*^2^* = 13.41, *p* < .001) and Target Letter Identity × Grade (χ*^2^* = 85.47, *p* < .001). For the variables of interest, there was a main effect of Grade (χ*^2^* = 24.21, *p* < .001), which was further modulated by a two-way interaction between Grade and Position (χ*^2^* = 26.49, *p* < .001), indicating that letters in the reversed position were recognized more correctly than in the regular position by 3^rd^-graders (*z* = 2.922, *p* = 0.004), but not by 5^th^-graders (*z* = 0.707, *p* = 0.480). For adults, the effect was opposite: letters were recognized more correctly in the regular position than in the reversed position (*z* = –4.250, *p* < .001). The full model output is presented in the Supplementary Material Table S4.

For the analysis of the response times, incorrect responses were removed (3^rd^ grade: 20.31%, 5^th^ grade: 16.83%, adults: 11.05%), and response times below 200ms were removed as false alarms (3^rd^ grade: 0.08%, 5^th^ grade: 0.06%, adults: 0.04%). After logarithmically transforming the response times, further outliers were trimmed by removing all data points with residuals exceeding 2.5 SD based on a simple model (3^rd^ grade: 2.34%, 5^th^ grade: 2.18%, adults: 2.07%). The final linear-mixed effects model revealed significant effects of the covariates Trial Order × Grade (χ*^2^* = 158.43, *p* < .001) and Target Letter Identity × Grade (χ*^2^* = 290.33, *p* < .001). For the variables of interest, we found a main effect of Grade (χ*^2^* = 122.93, *p* < .001) and a main effect of Position (χ*^2^* = 8.14, *p* = .004). Those main effects entered into a two-way interaction of Grade and Position (χ*^2^* = 21.04, *p* < .001), indicating that responses were faster in the reversed position for 3^rd^-graders (*z* = 3.996, *p* < 0.001), as well as for 5^th^-graders (*z* = 2.292, *p* = 0.022), but not for adults, where the trend was in the other direction, but did not reach significance (*z* = –1.708, *p* = 0.088). The full model output is presented in the Supplementary Material Table S5.

## Discussion

The results of our self-replication attempt diverge somewhat from our original results. The analysis of error rates suggests that 3^rd^-grade children are overall better at detecting a letter in the reversed position as compared to the regular position. This is in line with findings from Experiment 1 and is most likely due to more left-to-right than parallel processing of the letter string in the younger readers, as also Antzaka et al. (2020) observed for Basque children. In our study, the left-to-right bias seems to vanish by grade 5. Interestingly, it is opposite in adults, which were better at detecting a letter in the regular position at the end of the string. However, even in adults, there was no difference between suffixes and non-suffixes in their regular position, as we found in Experiment 1 and would have expected if suffixes were processed as position-specific units. The same picture emerges from the response time analysis: while we find the younger children to be faster in detecting a letter in the reversed than the regular position, probably due to a left-to-right processing bias, we do not find an effect of or any interaction with Affix Status, thus not providing any evidence for suffixes being processed as position-specific units.

It is worthwhile to recall that Beyersmann et al. (2015) found inhibitory effects of affix status on letter detection only for suffixed vs. non-suffixed pseudowords (e.g., *R* in *film**ure*** vs. *R* in *film**ire***), but also a numerical (i.e., not statistically significant) facilitation for prefixed compared to non-prefixed items (e.g., *R* in ***pro**point* vs. *R* in ***cro**point*). They suggest the tentative hypothesis that the inhibitory effect for suffixes, which is in line with results from letter search in multi-letter graphemes (Brand et al., 2007; Rey et al. 2000) reflects the sublexical status of suffixes. The non-significant facilitation for prefixes, by contrast, was suggested to reflect the “quasi-lexical” status of prefixes, eliciting facilitation much weaker than the word superiority effect of completely lexical words (e.g., Reicher, 1969; Wheeler, 1970). Our results for Italian suffixes in Experiment 2 are in line with the pattern Beyersmann et al. found for prefixes in French. Following Beyersmann’s reasoning, this would mean that Italian suffixes can be considered quasi-lexical, neither eliciting true sublexical nor true lexical effects. This could explain why we observed rather unstable effects of Affix Status in Experiment 1 and null effects in Experiment 2. According to some linguistic analyses, Italian is more morphologically rich than French (for a review see Borleffs, Maassen, Lyytinen & Zwarts, 2017). However, recent evidence from reading aloud suggests no morphological processing differences between French and Italian in developing or skilled readers (Mousikou et al., 2020). Hence, there is little reason to believe that suffixes are fundamentally different processing units in those two languages.

In order to have more power and gain a more comprehensive picture, we combined the data of Experiment 1 and 2 in a next step and analyzed them together. Additionally, because it is not possible with traditional frequentist null-hypothesis significance testing (NHST) to draw reliable conclusions from non-significant results (Dienes, 2014), such as the lack of the three-way interaction of Grade × Position × Affix Status in Experiment 2, we decided to also perform Bayes Factors analyses to investigate this interaction.

## Combined Analysis of Experiment 1 and 2

**Figure 5:**
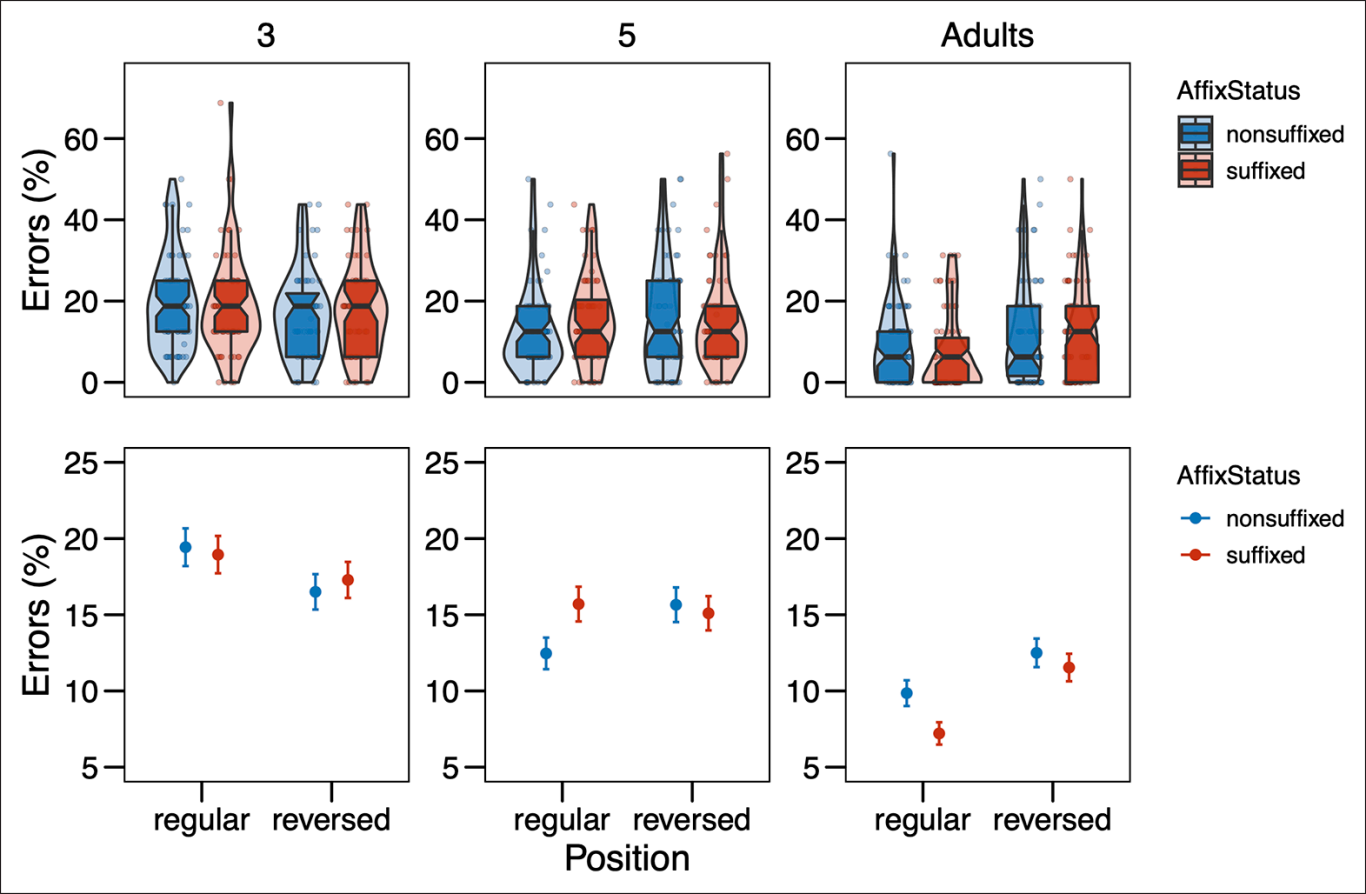
Error rates (in %) from Experiment 1 and 2 combined in the different conditions (Affix Status × Position) by age group. The upper row shows the distribution of the data, with boxplots indicating medians and interquartile ranges; the points represent by-subject means. In the bottom row, the points refer to the means by condition, while the error bars show the standard errors of the mean calculated at the trial-level.

**Figure 6:**
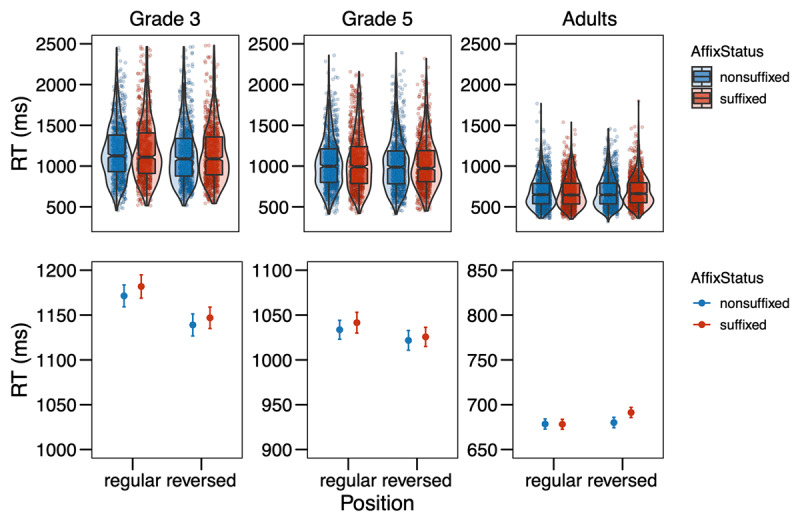
Response times (in ms) from Experiment 1 and 2 combined in the different conditions (Affix Status × Position) by age group. The upper row shows the distribution of the data, with boxplots indicating medians and interquartile ranges; the points represent single data points. In the bottom row, the points refer to the means by condition, while the error bars show the standard errors of the mean calculated at the trial-level. Note that the scales are equal across grades in the upper row, but range in the bottom row.

Means of error rates and response times from both experiments combined are presented in ***Table 3*** and ***Figures 5*** and ***6***, respectively. For the combined analysis, we used the same procedure with linear mixed-effects models as for the separate analyses (data and scripts available at *https://osf.io/yvtna/*). As for Experiment 1, a forward model selection procedure was used starting with Affix Status, Position, and Grade and their interactions as fixed effects as well as Participant and Item as random effects. Moreover, we added Experiment (1 vs. 2) as a random effect. Trial Order and Target Letter Identity and their interaction with Grade were added as fixed effects when model comparison suggested that they significantly improved the model fit.

**Table 3:** Means and ΔMeans of Error Rates and Response Times in Experiment 1 and 2 combined (Standard Deviations in Parentheses).


POSITION	REGULAR			REVERSED		

AFFIX STATUS	SUFFIXED	NONSUFFIXED	Δ_(NON-SUFF)_	SUFFIXED	NONSUFFIXED	Δ_(NON-SUFF)_

Error Rates (in %)						

Grade 3	18.95 (1.22)	19.43 (1.24)	0.48	17.29 (1.18)	16.50 (1.16)	–0.79

Grade 5	15.70 (1.14)	12.46 (1.04)	–3.24	15.10 (1.12)	15.66 (1.14)	0.56

Adults	7.21 (0.73)	9.86 (0.84)	2.65*	11.54 (0.90)	12.50 (0.94)	0.96*

Response Times (in ms)						

Grade 3	1182 (370)	1171 (345)	–11	1147 (345)	1139 (356)	–8

Grade 5	1042 (336)	1034 (313)	–8	1026 (311)	1022 (321)	–4

Adults	678 (187)	679 (188)	1	691 (186)	680 (189)	–11


* Significant difference (*p* < .05) according to the reported linear mixed-effects model.

For the analysis of the error rates, the final model included Affix Status, Position, Grade, and their interactions as fixed effects, as well as Trial Order and an interaction of Target Letter Identity and Grade as covariates, and Participant, Item, and Experiment as random effects. The model revealed significant effects of the covariates Trial Order (χ*^2^* = 28.22, *p* < .001) and Target Letter Identity × Grade (χ*^2^* = 181.98, *p* < .001). For the variables of interest, there was a main effect of Grade (χ*^2^* = 44.33, *p* < .001), which was further modulated by a two-way interaction between Grade and Position (χ*^2^* = 21.01, *p* < .001), indicating that letters in the reversed position and the regular position were similarly error prone in 3^rd^-graders (*z* = 1.96, *p* = 0.051) and 5^th^-graders (*z* = –1.33, *p* = 0.183), whereas adults recognized letters more correctly in the regular position than in the reversed position (*z* = –4.18, *p* < .001). There was also a two-way interaction between Grade and Affix Status (χ^2^ = 7.30, *p* = .026), indicating letters in suffixed and non-suffixed items were similarly error-prone in 3^rd^-graders (*z* = 0.16, *p* = 0.872) and 5^th^-graders (*z* = 1.366, *p* = 0.172), whereas adults recognized letters more correctly in suffixed than in non-suffixed items (*z* = –2.27, *p* = .023) Finally, the three-way interaction of Affix Status, Position, and Grade did not reach significance, even though it came close (χ^2^ = 5.68, p = .059). The full model output is presented in the Supplementary Material Table S6.

For the analysis of the response times, incorrect responses were removed (3^rd^ grade: 18.04%, 5^th^ grade: 14.73%, adults: 10.28%), and response times below 200ms were removed as false alarms (3^rd^ grade: 0.06%, 5^th^ grade: 0.03%, adults: 0.02%). After logarithmically transforming the response times, further outliers were trimmed by removing all data points with residuals exceeding 2.5 SD based on a simple model (3^rd^ grade: 1.91%, 5^th^ grade: 1.87%, adults: 2.17%). The final linear-mixed effects model revealed significant effects of the covariates Trial Order × Grade (χ*^2^* = 284.54, *p* < .001) and Target Letter Identity × Grade (χ*^2^* = 524.98, *p* < .001). For the variables of interest, we found a main effect of Grade (χ*^2^* = 303.12, *p* < .001) and a main effect of Position (χ*^2^* = 12.52, *p* < .001). Those main effects entered into a two-way interaction of Grade and Position (χ*^2^* = 22.57, *p* < .001), indicating that responses were faster in the reversed position for 3^rd^-graders (*z* = 4.78, *p* < 0.001), as well as for 5^th^-graders (*z* = 2.76, *p* = 0.006), but not for adults (*z* = –1.14, *p* = 0.254). The full model output is presented in the Supplementary Material Table S7.

In addition to the linear mixed-effects models, we conducted Bayes Factor analyses (for an introduction, see Schmalz, Biurrun Manresa, & Zhang, 2020) using the R package BayesFactor (Morey & Rouder, 2014; Morey et al., 2018) to test the three-way interaction of Grade × Position × Affix Status and the two-way interaction of Position × Affix Status. Those were the interactions that were at the center of our research question, but turned out to be rather unstable in our mixed-effects model analyses. For the analysis presented here, we used the default prior of the BayesFactor package (i.e., a Cauchy distribution centered around zero with a width of 0.707). In the Supplementary Material, we additionally report a sensitivity analysis using different priors to ensure that our results persist. For the error rate data, we used the final model (including the 3-way interaction of Grade × Position × Affix Status) and compared it to a model without this interaction. The analysis yielded a BF close to zero (BF10 = 0.029 +/– 5%), which, according to the classification by Lee and Wagenmakers (2013), can be counted as “very strong” evidence against a model including the three-way interaction. Moreover, we compared the model with all the two-way interactions to a model without the Position × Affix Status interaction, yielding again a BF close to zero (BF10 = 0.027 +/– 5%), thus indicating very strong evidence against the inclusion of this two-way interaction.

For the response time analysis, we followed the same procedure: we compared the full final model to a model without the three-way interaction, using the default prior. This resulted in a BF providing very strong evidence against the 3-way interaction (BF10 = 0.006 +/– 8%). Next, we compared the model with all two-way interactions to a model without the Position x Affix Status interaction, again yielding a BF close to zero (BF10 = 0.043 +/– 6%), thus indicating strong evidence against this interaction.

## General Discussion

The present study used a letter-search task with suffixed and non-suffixed pseudowords to investigate the early visuo-orthographic and position-specific processing of suffixes in Italian developing and skilled readers. Similar to a previous study by Beyersmann et al. (2015), participants in our study were asked to detect a target letter either in the suffix of a pseudoword (e.g., *S* in *flag**ish***) or in a nonsuffix control (e.g., *S* in *flag**osh***). In order to investigate sensitivity to morpheme position, we extended the paradigm to pseudowords with reversed morphemes: letters also had to be detected in affixes in their untypical position (e.g., *S* in ***ish**flag*) with non-suffixed pseudowords as controls (e.g., *S* in ***osh**flag*). This was grounded in the idea that suffixes, if identified in a position-specific fashion, should not be automatically perceived as units in the reversed condition, and consequently any difference between suffixes and non-suffixes in the regular position should disappear in the reversed position.

Based on the evidence in the literature, we assumed that the letter search task should be sensitive to the morphological status of the stimuli, at least for skilled readers: letters belonging to units identified as suffixes by the readers (e.g., *ish* in *flag**ish***) would behave differently from letters lacking this feature (e.g., *flag**osh***). We further suggested two opposing hypotheses for the direction of the effect, either facilitation or inhibition from suffixes. Contrary to our expectations, we only found very weak evidence for the role of the morphological status in our first, pre-registered experiment and inconsistent evidence across error rates and response times for position-specificity with a pattern that did not fully fit any of the two suggested hypotheses. The second experiment did not yield any evidence for a role of morphological status in letter detection, neither for any kind of position-specificity. A combined analysis of the two experiments suggested an effect of morphological status, but not of position-specificity, only for adults in the error rates (fewer errors in suffixed than non-suffixed items), but not for children and not in response times. Finally, an additional Bayes Factor analyses indicated strong to very strong evidence against a modulating role of morphemes as position-specific units in a letter search task. Overall, the effects under investigation were rather weak and inconsistent across experiments.

Hence, our experiments showed no conclusive evidence for suffixes as visuo-orthographic units – neither as sublexical units that inhibit single letter activation, as observed for multi-letter graphemes (Brand et al., 2007; Rey et al. 2000), nor as lexical units that provide facilitatory lexical feedback, as observed for words (Reicher, 1969; Wheeler, 1970). As we have discussed above, they could at the most be interpreted as “quasi-lexical” units, following Beyersmann et al.’s (2015) suggestion. However, there are no converging empirical or theoretical studies that make a strong case for assuming such a cross-linguistic difference between Italian and French and it seems at odds with previous research.

Another reason for the convoluted results could lie in interindividual variability. There is evidence from masked priming that individuals use different strategies in morphological processing (Andrews & Lo, 2013; Beyersmann, Casalis, Ziegler, & Grainger, 2014), which could have also been the case in the present task, leading to weak and inconsistent overall effects. For example, some individuals might benefit from top-down activation of suffixes, whereas others might experience inhibition due to chunking. This type of individual differences is especially inflated when investigating reading development in children, who are generally more variable and diverse in their word processing. We present some explorations of interindividual differences in the Supplementary Material. They do indicate high variability in the effects, but no clear pattern that would lead us to strongly believe in systematic differences that might have obscured overall effects.

Consequently, a key conclusion from the present study is that the letter search task does not appear to be appropriate for probing morphological processing. The search process that is at the core of the letter search task might give rise to task-specific strategies that are rather different from recognizing a written word during natural reading. This might explain why effects of morphemes, and even position-specific effects, have been proven rather stable in lexical decision tasks (e.g., Crepaldi et al., 2010), but could not be reliably found in letter search tasks. What might be a more appropriate task to examine developing readers’ sensitivity to morphemes at early visuo-orthographic stages? A potentially useful alternative might be the same-different task, in which participants are presented with a referent stimulus and a target stimulus and are asked to decide whether they are identical or different. This task has been successfully used to probe orthographic processing during visual word recognition, while minimizing lexical influences (e.g., Massol, Duñabeitia, Carreiras, & Grainger, 2013; Schmalz, Mulatti, & Job, 2020). Such a task could be implemented to test whether a letter change is easier or harder to detect in suffixes vs. nonsuffixes (e.g., *flagith – flagish, flagoth - flagosh*). The decision could still be based entirely on visual information, but without initiating search strategies by drawing attention to a single letter. Testing this empirically is an interesting endeavor for follow-up research.

A finding that was very consistent across Experiment 1 and 2 and the combined analysis, however, was the role of position that changed across development. It became very clear that developing readers scan the string serially, thus having a strong left-to-right bias in their target letter detection. This is in accordance with the finding from Antzaka et al. (2020) and with other previous studies that provided evidence that developing readers of transparent orthographies process letter strings in letter search tasks in a rather serial (left-to-right) fashion (e.g., Ktori & Pitchford, 2008, 2009). While this finding does not speak to the issue we set out to investigate, namely the position-specific visuo-orthographic processing of suffixes, it does affirm the wide-spread assumption that reading acquisition evolves from serial to more parallel processing (e.g., Grainger & Ziegler, 2011; Share, 1995).

Our example of a failed self-replication illustrates that we need to interpret small effects with caution and not draw hasty conclusions. This links to the current discussion about the replication crisis in psychology (e.g., Ioannidis, 2005). Typical effects in psychology are small and statistical power is often low (e.g., Stevens & Brysbaert, 2016) – a combination at danger of leading to uninterpretable null-results or to overestimation of significant effects that cannot be replicated (Gelman & Carlin, 2014; see also Nicenboim, Vasishth, Engelmann, & Suckow. 2018). This is problematic if one wants to draw informative conclusions about effects to build future work or theories on. A number of measures have been suggested to improve this situation (e.g., Chambers, 2017). Attempting to replicate an effect found in one’s own study is one very useful option, as Nicenboim et al. (2018) illustrate. Other recommended measures are use of pre-registration, openly sharing data and analysis code, and moving away from NHST towards Bayesian data analysis methods (Chambers, 2017; Nicenboim et al., 2018). Our repeated failure to find clear effects in the present study can serve as a warning for psycholinguists that unexpected results should undergo a replication attempt before being interpreted as strong evidence and integrated into a theoretical framework post-hoc. If we had done so after the first experiment, we would have come to untenable and misleading conclusions.

## Data Accessibility Statement

Data and analysis scripts are accessible under *https://osf.io/yvtna/*.

## Additional File

The additional file for this article can be found as follows:

10.5334/joc.153.s1 Supplementary material 1. Additional information regarding materials and results and additional exploratory analyses.

## Footnotes

Note that here and in other stimuli examples throughout the manuscript, we use bold font to differentiate affixes or non-affix-endings from stems. Bold font was not actually used in the presentation of the stimuli in any of the relevant experiments.

Note that this exclusion decision was made based on the distribution of the error rates, as stated in the preregistration. Hence, it was conditional on having seen the data, but before analyzing it, that is, blind to the pattern of results that might follow from this decision.

We note that the preregistration was not clear with respect to covariates, as we did not specify whether we would only add main effects or also interactions. We did use interactions in the model selection procedure. The rationale for entering Target Letter Identity and Grade as an interaction term was the idea that some letters might have been more perceptually salient due to their shape or familiarity to beginning readers.
