# Supplementary material for: Morpheme Position Coding in Reading Development as Explored With a Letter Search Task
Source: J Cogn. 2021 Feb 17;4(1):16. doi: 10.5334/joc.153 (PMC7894372; doi:10.5334/joc.153)
Supplement: Supplementary material 1. — Additional information regarding materials and results and additional exploratory analyses. [file joc-4-1-153-s1.pdf]

Supplementary Material

Table S1. Complete list of target stimuli used in the experiments.

Target Letter	Suffix	Non-suffix	Stem	Suffixed Regular	Nonsuffixed Regular	Suffixed Reversed	Nonsuffixed Reversed
L	ale	ole	vetro	vetrale	vetrole	alevetro	olevetro
L	ale	ole	tasca	tascale	tascole	aletasca	oletasca
L	ale	ole	acqua	acquale	acquole	aleacqua	oleacqua
L	ale	ole	cibo	cibale	cibole	alecibo	olecibo
L	ale	ole	carta	cartale	cartole	alecarta	olecarta
L	ale	ole	zuppa	zuppale	zuppole	alezuppa	olezuppa
L	ale	ole	treno	trenale	trenole	aletreno	oletreno
L	ale	ole	ponte	pontale	pontole	aleponte	oleponte
T	uto	oto	modo	moduto	modoto	utomodo	otomodo
T	uto	oto	nome	nomuto	nomoto	utonome	otonome
T	uto	oto	firma	firmuto	firmoto	utofirma	otofirma
T	uto	oto	legge	legguto	leggoto	utolegge	otolegge
T	uto	oto	palla	palluto	palloto	utopalla	otopalla
T	uto	oto	spada	spaduto	spadoto	utospada	otospada
T	uto	oto	pausa	pausuto	pausoto	utopausa	otopausa
T	uto	oto	naso	nasuto	nasoto	utonaso	otonaso
M	ame	eme	fonte	fontame	fonteme	amefonte	emefonte
M	ame	eme	letto	lettame	letteme	ameletto	emeletto
M	ame	eme	punto	puntame	punteme	amepunto	emepunto
M	ame	eme	festa	festame	festeme	amefesta	emefesta
M	ame	eme	barca	barcame	barceme	amebarca	emebarca
M	ame	eme	vento	ventame	venteme	amevento	emevento
M	ame	eme	sacco	saccame	sacceme	amesacco	emesacco
M	ame	eme	croce	crocame	croceme	amecroce	emecroce
S	oso	eso	parte	partoso	parteso	osoparte	esoparte
S	oso	eso	notte	nottoso	notteso	osonotte	esonotte
S	oso	eso	corda	cordoso	cordeso	osocorda	esocorda
S	oso	eso	libro	libroso	libreso	osolibro	esolibro
S	oso	eso	banca	bancoso	banceso	osobanca	esobanca
S	oso	eso	torta	tortoso	torteso	osotorta	esotorta
S	oso	eso	fiume	fiumoso	fiumeso	osofiume	esofiume
S	oso	eso	lupo	luposo	lupeso	osolupo	esolupo
N	enza	enta	gioco	giocenza	giocenta	enzagioco	entagioco
N	enza	enta	roba	robenza	robenta	enzaroba	entaroba
N	enza	enta	posta	postenza	postenta	enzaposta	entaposta
N	enza	enta	passo	passenza	passenta	enzapasso	entapasso

N	enza	enta	tazza	tazzenza	tazzenta	enzatazza	entatazza
N	enza	enta	borsa	borsenza	borsenta	enzaborsa	entaborsa
N	enza	enta	lago	lagenza	lagenta	enzalago	entalago
N	enza	enta	testa	testenza	testenta	enzatesta	entatesta
S	ismo	isco	cane	canismo	canisco	ismocane	iscocane
S	ismo	isco	luce	lucismo	lucisco	ismoluce	iscoluce
S	ismo	isco	gatto	gattismo	gattisco	ismogatto	iscogatto
S	ismo	isco	cena	cenismo	cenisco	ismocena	iscocena
S	ismo	isco	tetto	tettismo	tettisco	ismotetto	iscotetto
S	ismo	isco	rete	retismo	retisco	ismorete	iscorete
S	ismo	isco	torre	torrismo	torrisco	ismotorre	iscotorre
S	ismo	isco	campo	campismo	campisco	ismocampo	iscocampo
R	ario	arlo	amico	amicario	amicarlo	arioamico	arloamico
R	ario	arlo	neve	nevario	nevarlo	arioneve	arloneve
R	ario	arlo	fame	famario	famarlo	ariofame	arlofame
R	ario	arlo	luogo	luogario	luogarlo	arioluogo	arloluogo
R	ario	arlo	fuoco	fuocario	fuocarlo	ariofuoco	arlofuoco
R	ario	arlo	paese	paesario	paesarlo	ariopaese	arlopaese
R	ario	arlo	pugno	pugnario	pugnarlo	ariopugno	arlopugno
R	ario	arlo	pezzo	pezzario	pezzarlo	ariopezzo	arlopezze
S	ista	osta	muro	murista	murosta	istamuro	ostamuro
S	ista	osta	burro	burrista	burrosta	istaburro	ostaburro
S	ista	osta	porto	portista	portosta	istaporto	ostaporto
S	ista	osta	nave	navista	navosta	istanave	ostanave
S	ista	osta	cuore	cuorista	cuorosta	istacuore	ostacuore
S	ista	osta	pace	pacista	pacosta	istapace	ostapace
S	ista	osta	mondo	mondista	mondosta	istamondo	ostamondo
S	ista	osta	gara	garista	garosta	istagara	ostagara

Table S2. Model output from the analysis of the accuracy data of Experiment 1. χ^2-, z-, and p-values for fixed effects were calculated using Type 3 Wald Chi-square tests. Post-hoc contrasts were calculated using multiple pairwise comparisons.

Fixed Effects					
	χ^2	Est/Beta	SE	z	p
Intercept	825.62	2.03	0.07	28.73	<0.001
Affix Status	< 1	-0.03	0.04	-0.67	0.504
Position	2.73	0.06	0.04	1.65	0.099
Grade	21.92				<0.001
Affix Status X Position	< 1	-0.02	0.04	-0.39	0.699
Affix Status Regular		-0.08	0.11	-0.72	0.469
Affix Status Reversed		-0.02	0.11	-0.21	0.84
Affix Status X Grade	3.87				0.145
Affix Status Grade 3		-0.05	0.11	-0.47	0.640
Affix Status Grade 5		0.14	0.14	1.05	0.295
Affix Status Adults		-0.25	0.15	-1.69	0.091
Position X Grade	2.26				0.324
Position Grade 3		-0.02	0.11	-0.16	0.875
Position Grade 5		0.16	0.14	1.19	0.233
Position Adults		0.24	0.15	1.65	0.098
Affix Status X Position X Grade	9.42				0.009
Affix Status Regular Grade 3		-0.06	0.16	-0.37	0.709
Affix Status Reversed Grade 3		-0.05	0.16	-0.29	0.773
Affix Status Regular Grade 5		0.41	0.20	2.04	0.042
Affix Status Reversed Grade 5		-0.12	0.19	-0.64	0.524
Affix Status Regular Adults		-0.59	0.22	-2.73	0.006
Affix Status Reversed Adults		0.10	0.20	0.51	0.610
Trial Count	14.04	0.14	0.04	3.75	<0.001
Target Letter Identity X Grade	123.38				<0.001

Random Effects		
	Variance	S.D.
Participant (Intercept)	0.33	0.58
Item (Intercept)	0.02	0.14

Model fit		
R^2	Marginal	Conditional
	0.07	0.16

Key: p-values for fixed effects calculated using Type III Wald Chi-square tests.
Pairwise comparisons computed using emmeans().
Confidence Intervals have been calculated using the Wald method.
Model equation: Accuracy ~ Affix * Position * Grade + Trial Order + Target Letter : Grade + (1 | Participant) + (1 | Item)

Table S3. Model output from the analysis of the response time data (final model after cleaning) of Experiment 1. χ^2-, z-, and p-values for fixed effects were calculated using Type 3 Wald Chi-square tests. Post-hoc contrasts were calculated using multiple pairwise comparisons.

Fixed Effects					
	χ^2	Est/Beta	SE	t/z	p
Intercept	139649	6.77	0.02	373.70	<0.001
Affix Status	3.63	-0.005	0.003	-1.90	0.057
Position	9.54	0.009	0.003	3.09	0.002
Grade	184.58				<0.001
Affix Status X Position	1.33	0.003	0.003	1.15	0.248
Affix Status Regular		-0.004	0.008	-0.53	0.595
Affix Status Reversed		-0.017	0.008	-2.16	0.031
Affix Status X Grade	< 1				0.655
Affix Status Grade 3		-0.008	0.009	-0.90	0.371
Affix Status Grade 5		-0.017	0.010	-1.80	0.072
Affix Status Adults		-0.006	0.009	-0.72	0.473
Position X Grade	13.68				0.001
Position Grade 3		0.037	0.009	4.07	<0.001
Position Grade 5		0.021	0.010	2.25	0.025
Position Adults		-0.007	0.009	-0.85	0.398
Affix Status X Position X Grade	8.55				0.014
Affix Status Regular Grade 3		-0.020	0.01	-1.58	0.114
Affix Status Reversed Grade 3		0.004	0.01	0.32	0.752
Affix Status Regular Grade 5		-0.009	0.01	-0.69	0.489
Affix Status Reversed Grade 5		-0.025	0.01	-1.85	0.065
Affix Status Regular Adults		0.017	0.01	1.42	0.156
Affix Status Reversed Adults		-0.029	0.01	-2.41	0.016
Trial Count X Grade	138.10				<0.001
Target Letter Identity X Grade	338.56				<0.001
Random Effects					
			Variance		S.D.
Participant (Intercept)			0.04		0.19
Item (Intercept)			<0.01		0.02
Model fit					
R^2			Marginal		Conditional
			0.46		0.72

Key: p-values for fixed effects calculated using Type III Wald Chi-square tests.
Pairwise comparisons computed using emmeans().
Confidence Intervals have been calculated using the Wald method.
Model equation: log(RT) ~ Affix * Position * Grade + Trial Order + Target Letter : Grade + (1 | Participant) + (1 | Item)

Table S4. Model output from the analysis of the accuracy data of Experiment 2. χ^2-, z-, and p-values for fixed effects were calculated using Type 3 Wald Chi-square tests. Post-hoc contrasts were calculated using multiple pairwise comparisons.

Fixed Effects					
	χ^2	Est/Beta	SE	z	p
Intercept	450.55	1.81	0.09	21.23	<0.001
Affix Status	< 1	0.01	0.04	0.15	0.880
Position	1.57	0.05	0.04	1.25	0.210
Grade	24.21				<0.001
Affix Status X Position	< 1	-0.001	0.04	0.05	0.961
Affix Status Regular		0.015	0.11	0.14	0.900
Affix Status Reversed		0.008	0.11	0.07	0.941
Affix Status X Grade	3.50				0.174
Affix Status Grade 3		0.11	0.13	0.81	0.420
Affix Status Grade 5		0.12	0.13	0.96	0.339
Affix Status Adults		-0.19	0.14	-1.39	0.164
Position X Grade	26.49				<0.001
Position Grade 3		-0.38	0.13	-2.92	0.004
Position Grade 5		0.09	0.13	0.71	0.480
Position Adults		0.58	0.14	4.25	<0.001
Affix Status X Position X Grade	1.78				0.410
Affix Status Regular Grade 3		-0.03	0.17	-0.17	0.862
Affix Status Reversed Grade 3		0.24	0.19	1.25	0.212
Affix Status Regular Grade 5		0.21	0.18	1.15	0.251
Affix Status Reversed Grade 5		0.03	0.18	0.19	0.846
Affix Status Regular Adults		-0.13	0.21	-0.62	0.537
Affix Status Reversed Adults		-0.25	0.17	-1.44	0.149
Trial Count	13.41	0.14	0.04	3.66	<0.001
Target Letter Identity X Grade	85.47				<0.001

Random Effects		
	Variance	S.D.
Participant (Intercept)	0.51	0.71
Item (Intercept)	0.02	0.15

Model fit		
R^2	Marginal	Conditional
	0.08	0.20

Key: p-values for fixed effects calculated using Type III Wald Chi-square tests.
Pairwise comparisons computed using emmeans().
Confidence Intervals have been calculated using the Wald method.
Model equation: Accuracy ~ Affix * Position * Grade + Trial Order + Target Letter : Grade + (1 | Participant) + (1 | Item)

Table S5. Model output from the analysis of the response time data (final model after cleaning) of Experiment 2. χ^2-, z-, and p-values for fixed effects were calculated using Type 3 Wald Chi-square tests. Post-hoc contrasts were calculated using multiple pairwise comparisons.

Fixed Effects					
	χ^2	Est/Beta	SE	t/z	p
Intercept	101617	6.84	0.02	318.77	<0.001
Affix Status	< 1	-0.001	0.003	0.33	0.742
Position	8.14	0.009	0.003	2.85	0.004
Grade	122.93				<0.001
Affix Status X Position	< 1	-0.0004	0.003	-0.12	0.905
Affix Status Regular		0.001	0.009	0.15	0.882
Affix Status Reversed		0.003	0.009	0.32	0.750
Affix Status X Grade	3.29				0.193
Affix Status Grade 3		0.005	0.012	0.44	0.662
Affix Status Grade 5		0.012	0.010	1.17	0.244
Affix Status Adults		-0.011	0.009	-1.21	0.227
Position X Grade	21.04				<0.001
Position Grade 3		0.047	0.012	4.00	0.001
Position Grade 5		0.024	0.011	2.29	0.022
Position Adults		-0.015	0.009	-1.71	0.088
Affix Status X Position X Grade	1.40				0.497
Affix Status Regular Grade 3		0.014	0.01	0.86	0.390
Affix Status Reversed Grade 3		-0.004	0.02	-0.27	0.791
Affix Status Regular Grade 5		0.007	0.02	0.49	0.627
Affix Status Reversed Grade 5		0.017	0.02	1.16	0.246
Affix Status Regular Adults		-0.018	0.01	-1.40	0.163
Affix Status Reversed Adults		-0.004	0.01	-0.33	0.743
Trial Count X Grade	158.43				<0.001
Target Letter Identity X Grade	290.33				<0.001

Random Effects		
	Variance	S.D.
Participant (Intercept)	0.04	0.21
Item (Intercept)	<0.01	0.02

Model fit		
R^2	Marginal	Conditional
	0.41	0.71

Key: p-values for fixed effects calculated using Type III Wald Chi-square tests.
Pairwise comparisons computed using emmeans().
Confidence Intervals have been calculated using the Wald method.
Model equation: log(RT) ~ Affix * Position * Grade + Trial Order + Target Letter : Grade + (1 | Participant) + (1 | Item)

Table S6. Model output from the analysis of the accuracy data of Experiment 1 and 2 combined. χ^2-, z-, and p-values for fixed effects were calculated using Type 3 Wald Chi-square tests. Post-hoc contrasts were calculated using multiple pairwise comparisons.

Fixed Effects					
	χ^2	Est/Beta	SE	z	p
Intercept	186.46	1.52	0.11	13.66	<0.001
Affix Status	< 1	0.007	0.04	0.16	0.872
Position	3.82	-0.09	0.04	-1.96	0.051
Grade	44.33				<0.001
Affix Status X Position	< 1	-0.03	0.04	-0.70	0.483
Affix Status Regular		-0.04	0.08	-0.45	0.650
Affix Status Reversed		-0.02	0.08	-0.25	0.81
Affix Status X Grade	7.30				0.026
Affix Status Grade 3		0.01	0.09	0.16	0.872
Affix Status Grade 5		0.13	0.09	1.37	0.172
Affix Status Adults		-0.23	0.10	-2.27	0.023
Position X Grade	21.01				<0.001
Position Grade 3		-0.17	0.09	-1.96	0.051
Position Grade 5		0.13	0.09	1.33	0.18
Position Adults		0.42	0.10	4.18	<0.001
Affix Status X Position X Grade	5.68				0.059
Affix Status Regular Grade 3		-0.05	0.12	-0.39	0.696
Affix Status Reversed Grade 3		0.08	0.13	0.60	0.550
Affix Status Regular Grade 5		0.30	0.14	2.18	0.030
Affix Status Reversed Grade 5		-0.04	0.13	-0.29	0.768
Affix Status Regular Adults		-0.36	0.15	-2.37	0.018
Affix Status Reversed Adults		-0.10	0.13	-0.73	0.467
Trial Order	28.22	0.14	0.03	5.31	<0.001
Target Letter Identity X Grade	181.98				<0.001

Random Effects		
	Variance	S.D.
Participant (Intercept)	0.43	0.66
Item (Intercept)	0.03	0.18

Model fit		
R^2	Marginal	Conditional
	0.07	0.18

Key: p-values for fixed effects calculated using Type III Wald Chi-square tests.
Pairwise comparisons computed using emmeans().
Confidence Intervals have been calculated using the Wald method.
Model equation: Accuracy ~ Affix * Position * Grade + Trial Order + Target Letter : Grade + (1 | Participant) + (1 | Item)

Table S7. Model output from the analysis of the response time data (final model after cleaning) of Experiment 1 and 2 combined. χ^2-, z-, and p-values for fixed effects were calculated using Type 3 Wald Chi-square tests. Post-hoc contrasts were calculated using multiple pairwise comparisons.

Fixed Effects					
	χ^2	Est/Beta	SE	t/z	p
Intercept	48886	6.81	0.03	221.10	<0.001
Affix Status	1.99	-0.003	0.002	-1.41	0.158
Position	12.52	0.008	0.002	3.54	<0.001
Grade	303.13				<0.001
Affix Status X Position	< 1	-0.001	0.002	0.56	0.577
Affix Status Regular		-0.004	0.007	-0.60	0.546
Affix Status Reversed		-0.009	0.007	-1.39	0.164
Affix Status X Grade	< 1				0.862
Affix Status Grade 3		-0.006	0.008	-0.76	0.449
Affix Status Grade 5		-0.005	0.007	-0.61	0.544
Affix Status Adults		-0.009	0.007	-1.40	0.160
Position X Grade	22.57				<0.001
Position Grade 3		0.036	0.008	4.78	<0.001
Position Grade 5		0.020	0.007	2.76	0.006
Position Adults		-0.008	0.007	-1.14	0.254
Affix Status X Position X Grade	1.25				0.535
Affix Status Regular Grade 3		-0.007	0.01	-0.63	0.529
Affix Status Reversed Grade 3		-0.005	0.01	-0.44	0.660
Affix Status Regular Grade 5		-0.004	0.01	-0.40	0.689
Affix Status Reversed Grade 5		-0.005	0.01	-0.46	0.648
Affix Status Regular Adults		-0.0009	0.009	-0.09	0.927
Affix Status Reversed Adults		-0.018	0.01	-1.88	0.061
Trial Order X Grade	284.54				<0.001
Target Letter Identity X Grade	524.98				<0.001
Random Effects					
			Variance		S.D.
Participant (Intercept)			0.04		0.20
Item (Intercept)			<0.01		0.02
Experiment (Intercept)			<0.01		0.04
Model fit					
R^2			Marginal		Conditional
			0.42		0.71

Key: p-values for fixed effects calculated using Type III Wald Chi-square tests.
Pairwise comparisons computed using emmeans().
Confidence Intervals have been calculated using the Wald method.

Model equation: log(RT) ~ Affix * Position * Grade + Trial Order + Target Letter : Grade + (1 | Participant) + (1 | Item)

Bayes Factor sensitivity analysis of the combined data using different priors

One additional advantage of using BF analyses is that researchers can take into account their own expectations about possible effects based on previous similar studies and include those as priors. The only study that is more directly comparable to ours and thus can be used to derive informative priors is the one by Beyersmann et al. (2015). Despite the different language under investigation and small differences in the presentation procedure, the main difference between their and our study is that Beyersmann et al. do not have a „reversed" condition, as we do, but instead have a regular prefix condition, which we do not have. Moreover, they did not obtain data from different age groups, but only adults. Their important effect for comparison is the difference between suffixed and nonsuffixed items in word-final position. We calculate the effect size for both RT (d=0.143) and Error Rates (d=-0.048) based on the means and standard deviations that the authors provide in their article. There was no effect in the Error Rates and, as is typical for letter search paradigms, a rather small effect in the RTs. Based on the small effect sizes in the RTs, we reran our BF analyses for RTs with a range of smaller priors, assuming smaller expected effects than the default. This sensitivity analyses allows to investigate whether we continue to get support for the null effect even if we assume any true effect to be very small. We again used the Cauchy distribution centered around zero but changed the width parameter. There was still evidence for the null with values of 0.5, 0.3, 0.1, and 0.05. Results are summarized in Table S8.

Table S8. Results from BF analyses on RTs using different width parameters for the prior distribution.

Width Parameter of the Prior	0.5	0.3	0.1	0.05
BF10<br>Full Final Model / Model without 3-way Interaction	0.0022<br>(+/- 37.33%)	0.010<br>(+/- 37.89%)	0.084<br>(+/- 11.45%)	0.221<br>(+/- 12.12%)
BF10<br>Model with all 2-way Interactions / Model without Position x Affix Status Interaction	0.106<br>(+/- 37.29%)	0.106<br>(+/- 35.09%)	0.240<br>(+/- 12.98%)	0.455<br>(+/- 10.87%)

Interindividual differences

To check this idea post-hoc, we calculated the effect of affix status (response time difference between nonsuffixed and suffixed items: ΔRT) in both the regular and reversed position for each participant. Indeed, participants varied a lot in whether they showed faster responses for suffixed or nonsuffixed items in each position. Figure S1 illustrates this high interindividual variability: there are some participants that had faster response time for suffixes than nonsuffixes regardless of the position (upper right quarter of the plot), some participants that had slower response time for suffixes than nonsuffixes regardless of the position (lower left quarter of the plot), some participants that had faster response times for suffixes in the regular but slower ones in the reversed position (lower right quarter), and also some participants with slower response times for suffixes in the regular but faster ones in the reversed position (upper left quarter). The center point for each group is very close to zero, whereas the variability is disproportionally large. No convincing correlational pattern emerges (Grade 3: r=0.137; Grade 5: r=0.124; Adults: r=-0.080).

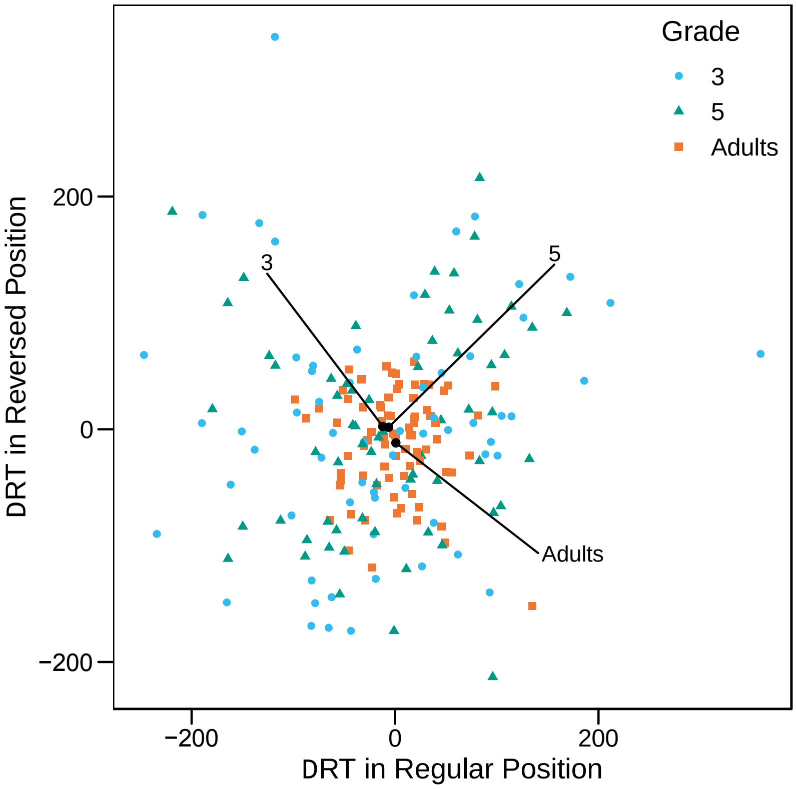

Figure S1. Effect of affix status (response time difference between nonsuffixed and suffixed items) in regular (x-axis) and reversed (y-axis) position for each participant. The center point (mean) for each group is indicated in black.

The previous studies indicating that individuals use different strategies in morphological processing have especially identified reading ability as an underlying factor for those differences (e.g., Andrews & Lo, 2013; Beyersmann et al., 2014, Hasenäcker et al. 2020). Unfortunately, we did not obtain any additional measures on reading ability or orthographic or morphological processing to test those assumptions in a principled manner. To nevertheless explore this possibility, we calculated a speed variable for each participant from their mean RTs and looked at the relationship with the ΔRT of affix status that we already described above (Figure S2). Correlations seem to indicate a weak negative relationship of reading speed and affix status in regular position for children (Grade 3: *r*=-0.251; Grade 5: *r*=-0.304), but not for adults (*r*=0.040). A weak positive relationship emerges in reversed position for 3rd-graders (r=0.273), but not for 5th-graders (*r*=0.060) or adults (*r*=0.080). This gives some indication that differences in reading ability might impact

morphological processing in developing readers. These should, however, not be overinterpreted, given the weakness of the correlations and the high variability in the children's data.

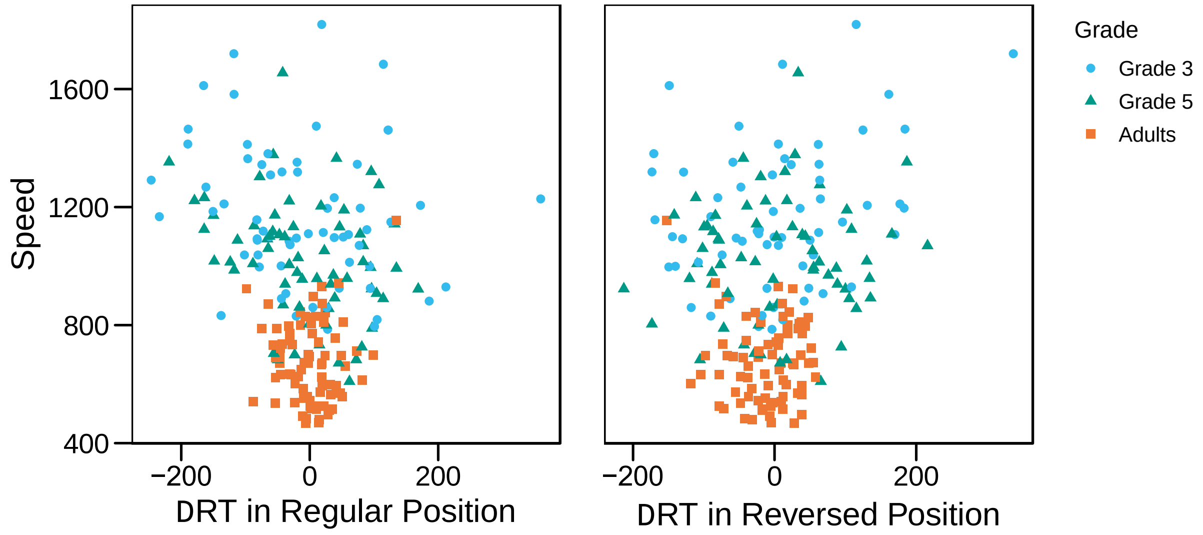

Figure S2. Speed (mean overall RT per participant; x-axis) and effect of affix status (response time difference between nonsuffixed and suffixed items; y-axis) in regular position (left panel) and reversed position (right panel) for each participant.
